# Characterization of a murine model of metastatic human non-small cell lung cancer and effect of CXCR4 inhibition on the growth of metastases

**DOI:** 10.18632/oncoscience.117

**Published:** 2015-02-09

**Authors:** Arvind K. Singla, Charlene M. Downey, Gwyn D. Bebb, Frank R. Jirik

**Affiliations:** ^1^ Department of Biochemistry and Molecular Biology, The McCaig Institute for Bone and Joint Health, Alberta, Canada; ^2^ Tom Baker Cancer Centre, and Department of Oncology, University of Calgary, Calgary, Alberta, Canada

**Keywords:** preclinical, NCI-H1299, metastasis model, NSCLC, AMD3100, CXCR4

## Abstract

Despite successful preclinical testing carried out through the use of subcutaneous xenografted tumors, many anti-cancer agents have gone on to fail in human trials. One potential factor accounting for this discrepancy may relate to the inadequacy of the commonly employed preclinical models to recapitulate the human disease, particularly when it comes to discovery of agents that are effective against advanced disease. Herein, we report the characterization of a NSCLC model and an exploration of the impact that a CXCR4 inhibitor, AMD3100, had on NCI-H1299-derived metastasis. These cells express a variety of metastasis-promoting factors, hence we selected them for a study of their metastatic colonization potential. To accomplish this, luciferase-expressing H1299 (H1299-luc2) cells were inoculated into athymic mice via the intracardiac route. This strategy produced adrenal, bone, ovarian, and pancreatic metastases, sites commonly involved in human metastatic NSCLC. Notably, micro-computed tomography and histological evaluation of the skeletal lesions revealed the presence of extensive osteolysis. To investigate the potential role of CXCR4 in mediating metastatic colonization of tissues, AMD3100 was administered to mice inoculated with H1299-luc2 cells. While this treatment did not appreciably alter the frequency of metastatic colonization, it was able to slow the growth of macrometastases. This model, recapitulating some of the events seen in late-stage human NSCLC, may prove useful in the evaluation of new therapies targeting metastatic disease.

## INTRODUCTION

At diagnosis, the majority of non-small cell lung cancer (NSCLC) patients present with advanced disease, and even when diagnosed at an early stage and treated surgically, cancer recurs in about one third of individuals, with eventual spread to lymph node, adrenal gland, bone, ovary, pancreas, liver, and brain [1]. Better treatments for advanced disease are clearly needed to combat this lethal cancer. Unfortunately, however, the majority of anticancer agents showing success at the pre-clinical testing stage have proceeded to fail in clinical trials [2, 3]. One of the many possible reasons for this high failure rate could be due to the discrepancy between the preclinical and clinical trial settings. For example, preclinical testing has most commonly relied on subcutaneously-implanted human NSCLC xenografts grown in immuno-compromised mice. Such models lack many of the features that are characteristic of the human disease, such as the development of metastases. In order to improve the success rate of anticancer drugs destined for use in human NSCLC, that the gap between preclinical and clinical studies must be narrowed via the establishment of models that more closely parallel the human disease.

Both genetically engineered mutant mice (GEMM) and human tumor cell line or patient-derived xenograft (PDX) models are being widely used in preclinical studies. Interestingly, GEMM appear to faithfully recapitulate tumor initiation and progression events, but are often relatively limited when it come to recapitulating late stage events, such as spontaneous metastasis [4]. Most studies of metastases carried out to date have employed xenograft models. Although distant metastases do appear in such models, the frequency of metastases can be low and unpredictable, and there is often the need to remove the primary xenograft by surgical means [5]. In addition, the majority of primary GEMM, or orthotopically-implanted xenografted lung tumors rarely metastasize, making studies of metastases relatively impractical. Thus, a more effective approach to generate systemic metastasis has relied on the administration of tumor cells directly into the arterial circulation, thus employing the ‘second half’ of the metastatic process: namely, extravasation, migration towards and colonization of a suitable microenvironmental niches, and development of macrometastases [4].

For a metastatic lesion to develop, the tumor cell must be able to interact with and recruit a variety of stromal cells, such as mesenchymal stem cells, cancer associated fibroblasts, endothelium, and various hematopoietic cell types [6, 7, 8]. Many of these events rely on the interactions between chemokines and their receptors. For example, the CXCL12/CXCR4 axis plays an important role in tumor cell extravasation and tissue-specific homing [9, 10]. Indeed, CXCR4 is widely expressed on tumor cells, and the preferential sites of metastases, such as bone, liver, and lung, express high levels of its ligand, CXCL12 [10].

Herein, we have developed a preclinical lung cancer metastasis model, based on the arterial (intra-cardiac) delivery of luciferase-expressing NCI-H1299 NSCLC cells. This method, which plausibly recapitulates the process of systemic metastatic colonization and growth, resulted in the development of metastases in a number of sites similar to those observed in humans. These included lesions of bone, adrenal gland, ovary, and pancreas. We found that although treatment with the CXCR4 inhibitor, AMD3100, attenuated metastatic growth, it failed to significantly impact the frequency of metastases, or overall survival of the mice. Preclinical models of late stage NSCLC, such as the one we report in this study, may prove instrumental for evaluating therapeutics aimed at advanced disease.

## RESULTS

### Establishing a metastasis model using H1299-luc2 cells

**Figure 1:**
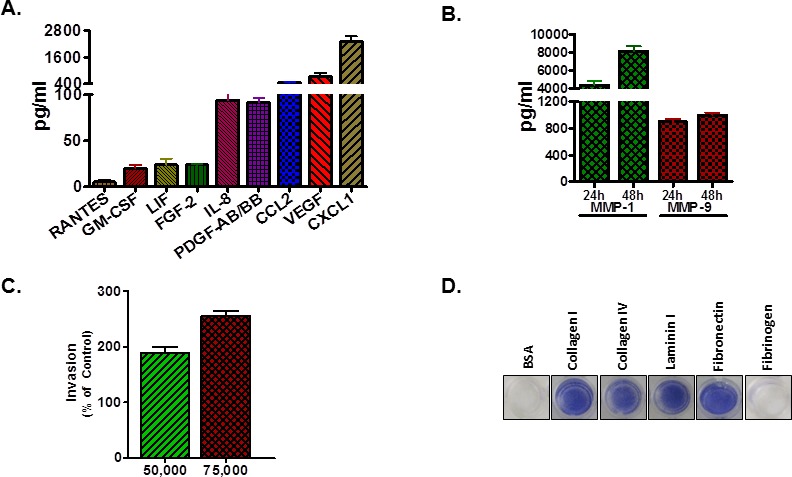
*In vitro* characterization of H1299-luc2 cells (A) Culture supernatants of H1299-luc2 cells contained RANTES (CCL5), GM-CSF, LIF, FGF-2, IL-8 (CXCL8), PDGF-AB/BB, MCP-1 (CCL2), VEGF and CXCL1 as determined by Luminex technology. (B) MMP-1 and MMP-9 production by H1299-luc2 cells. For A and B, results are shown as the mean ±SEM, n=4 biological replicates. (C) H1299–luc2 cells *in vitro* invasive ability, using 10% FBS as the chemoattractant. The mean number of invasive cells from five independent fields/well is indicated. (D) Representative images showing adhesion of H1299-luc2 cells to collagens I and IV, laminin I, and fibronectin.

A polyclonal population of H1299 cells, stably transfected with a CMV promoter-based vector for the expression of EGFP-luc2 fusion protein, were FACS sorted in order to obtain GFP^hi^ cells (Fig. Supplemental S1A). A fluorescence microscopy image demonstrates expression of EGFP in H1299-luc2 cells (Fig. Supplemental S1B), and the activity of luc2 showed a correlation between cell number and photon emission rates (Fig. Supplemental S1C). Human 65-plex cytokine/chemokine/growth factor and 8-plex MMP assay performed on H1299-luc2 cell supernatants revealed the presence of VEGF, IL-8 (CXCL8), CXCL1, MCP-1 (CCL2), PDGF-AB/BB, RANTES (CCL5), GM-CSF, LIF, FGF-2, MMP-1 and MMP-9 (Fig. 1A, 1B). Characterization of H1299-luc2 cell lysates using both western blotting and reverse phase protein array showed that the cells expressed CXCR4, EGFR, uPAR, N-cadherin, vimentin, as well as loss of PTEN and E-cadherin, and activation of the PI3K, MAPK/ERK, Stat3 and Src signal transduction pathways (data not shown), findings that were consistent with previous reports [11, 12, 13]. H1299-luc2 cells demonstrated an invasive phenotype through Matrigel in response to FBS stimulation, as well as an ability to adhere to the extracellular matrix proteins fibronectin, types I and IV collagen, and laminin I (Fig. 1C, D). Taken together, these results suggested that H1299-luc2 cells might possess a propensity for metastatic colonization.

### Characterization of the H1299-luc2 metastasis model

**Figure 2:**
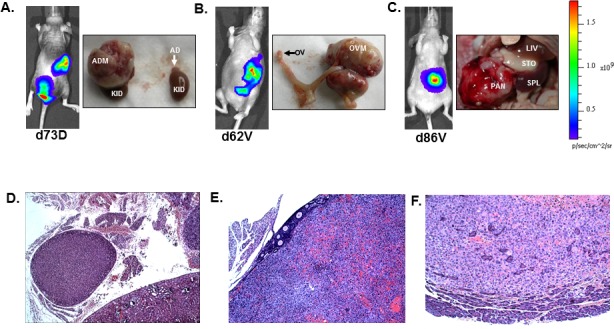
Soft tissue metastases following intracardiac inoculation of H1299-luc2 cells Representative BLI and corresponding gross anatomy images of adrenal (A), ovarian (B), and pancreatic (C), metastases. BLIs were captured at the indicated days (D, dorsal; V, ventral). Histopathology of adrenal (D), ovarian (E), and pancreatic (F) metastases. AD=adrenal, ADM= adrenal metastasis, KID=kidney, LIV=liver, OV=ovary, OVM=ovarian metastasis, PAN= pancreatic metastasis, SPL=spleen and STO=stomach.

**Figure 3:**
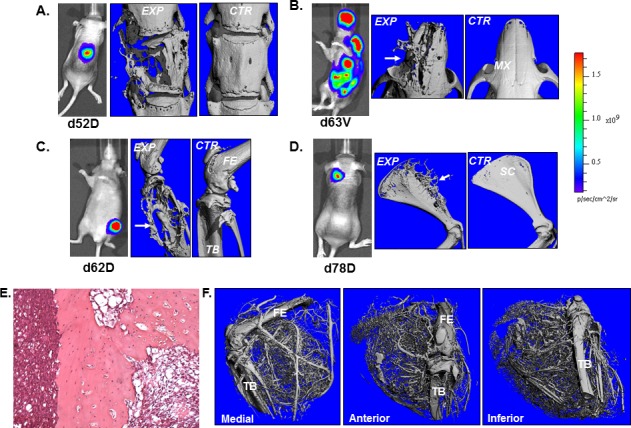
μCT imaging of skeletal metastases and tumor vasculature Representative BLI and μCT images of vertebral (A), maxillary (B), tibial (C) and scapular (D), metastasis. For each site, μCT images of the respective control bones is also shown. (E) Neovascular pattern in a metastasis that has escaped the confines of the bone as shown by μCT plus Microfil perfusion of the tumor vessels. Histopathology of bone metastasis (F). CTR=control bone, EXP=experimental, FE= femur, MX= maxilla, SC= scapula, TB=tibia and VT= vertebrae.

**Figure 4:**
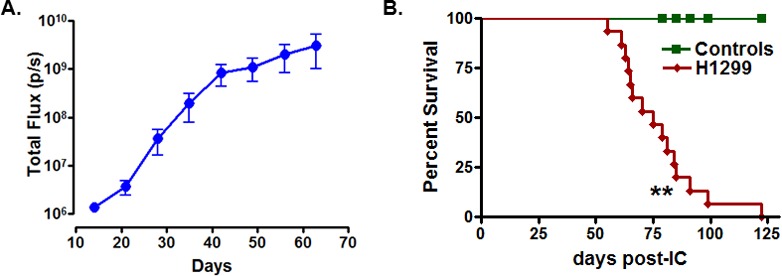
Site-specific incidence and growth rate of H1299-luc2 metastases, and impact on survival (A) Quantification of metastasis progression as shown by the increase in whole body photon emission rates over time. Data presented as mean ± SEM, rate is total flux (photons/sec). (B) Kaplan-Meier analysis showing decrease in survival for H1299-luc2 inoculated mice as compared to controls (log-rank test p = 0.0019, n=14, **p<0.01).

To evaluate the metastasis-inducing potential of H1299-luc2, 4×10^5^ cells were injected by either the intravenous or the intracardiac routes into NIH-III (*nude/nude*; *beige/beige*) outbred mice. Tumor development was monitored for up to 4 months *via* weekly bioluminescence imaging (BLI). H1299-luc2 cells inoculated *via* the intravenous route did not show lung or other organ metastasis for up to ~4 months (Fig. Supplemental S2A), in contrast, within 12 to 23 days after intracardiac injections of H1299-luc2 cells, BLI revealed the development of soft and bone-tissue macrometastases (Fig. 2, 3). Inspection and BLI of isolated organs, histopathology, and μCT revealed the presence of metastases at various anatomical sites, including adrenal gland (Fig. 2A), ovary (Fig. 2B), pancreas (Fig. 2C), and the skeleton (Fig. 3A-D). Table 1 shows the overall frequency of bone and soft-tissue metastases, and the increase in tumor load over time, is shown in Fig. 4A. Significant increases in metastatic burden (BLI signal ~10^9^ photons/sec) were associated with decreased survival (Fig. 4B). Notably, the spectrum of tissues involved by metastases in the mice echo those observed in human metastatic NSCLC, with the exception of liver and brain lesions that commonly occur in human NSCLC.

**Table 1:** Overall frequency and anatomical sites of metastasis generated by H1299-luc2 cells (n=14 mice with metastases)

Adrenal	Ovarian	Pancreatic	Maxillary/Mandibular	Femoral/Tibial	Vertebral	Scapular
2/14	2/14	1/14	3/14	3/14	3/14	2/14

### Metastasis histopathology and 3D μCT imaging of tumor neovasculature

Representative histopathology images showing adrenal gland (Fig. 2D), ovarian (Fig. 2E), pancreatic (Fig. 2F) and bone metastasis (Fig. 3E). The μCT images of bone metastatic lesions in mice that were perfused with Microfil, revealed evidence of extensive angiogenic response elicited by H1299-luc2 bone metastases that had spread from the bone into the adjacent soft tissues (Fig. 3F).

### CXCR4 inhibition reduces the growth of metastatic tumors without affecting the number of metastases or overall survival

**Figure 5:**
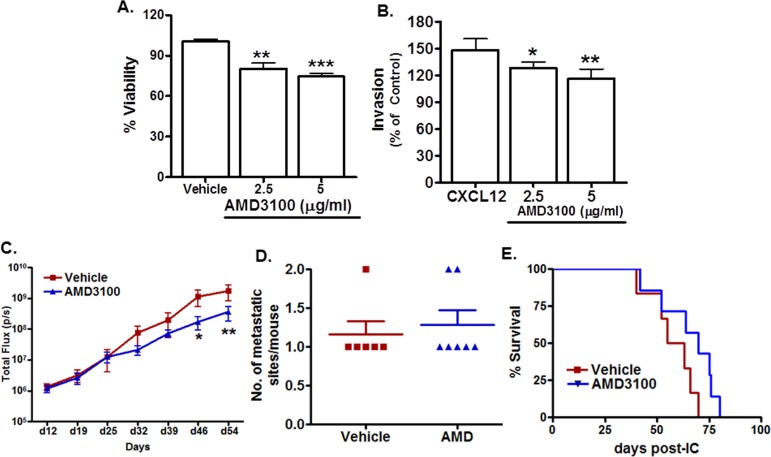
Effects of *in vitro* and *in vivo* CXCR4 inhibition on H1299-luc2 cells (A) MTT assay showed significantly decreased growth for H1299-luc2 cells cultured in presence of AMD3100 for 48 hrs (2.5 μg/ml; p=0.0041, 5 μg/ml; p<0.0001). (B) Reduced ***in vitro*** invasiveness by H1299-luc2 cells in presence of AMD3100 (2.5 μg/ml; p=0.035, 5 μg/ml; p= 0.0094). The *p* value was obtained using the unpaired two-tailed t test *p < 0.05, **p <0.01) (C) A line graph showing a comparison of metastatic colony growth between vehicle- and AMD3100-treated mice. There was a significant reduction in *in vivo* tumor growth in the treatment group, as compared to the vehicle control group. The *p* value was calculated by two way ANOVA with Bonferroni's post-test *p < 0.05 and **p<0.01. (F) Scatter dot plot showing the lack of a significant difference for total number of metastatic sites per mice between treatment and vehicle control groups of mice. Data are shown as the mean ±SEM. (G) Kaplan-Meier analysis showed no significant (p=0.1002) increase in overall survival for treatment group of mice compared to controls (log-rank test p = 0.1002).

We next determined whether CXCR4 inhibition with AMD3100 would limit the frequency of H1299-luc2 metastases. Immunoblotting confirmed that CXCR4 expression was present in H1299-luc2 cells (Fig. Supplemental S2B). Interestingly, *in vitro*, we found that AMD3100 treatment significantly suppressed the growth of H1299-luc2 cells as compared to controls (2.5 μg/ml; p=0.0041, 5 μg/ml; p<0.0001) (Fig. 5A), and also decreased the invasive ability of the cells in response to FBS (2.5 μg/ml; p=0.035, 5 μg/ml; p= 0.0094) (Fig. 5B), consistent with previous reports [14, 15]. The *in vivo* effect of AMD3100 (5 mg/kg daily) on metastatic colonization and/or growth was then evaluated as previously reported [16]. Importantly, BLI analysis revealed that AMD3100 treatment significantly reduced the growth of metastases, as compared to vehicle controls, at day 46 (p<0.05), and at day 54 (p<0.01) (Fig. 5C). AMD3100, however, did not appear to alter the frequency of H1299-luc2 macrometastases (Fig. 5D). Kaplan-Meier survival analysis showed only a marginal increase in overall survival in AMD3100 treated group of mice relative to controls (Fig. 5E). Thus, although CXCR4 inhibition neither impaired metastatic colonization nor significantly increased survival of the mice, it did have a negative effect on the growth of metastases, as determined by BLI.

## DISCUSSION

A plausible reason for the high failure rates of NSCLC anticancer drugs when moving from traditional preclinical models to the clinic may lie in the inability of the rodent models to recapitulate key features of the human disease, such as metastases [17]. Herein, we have characterized a metastasis model showing that H1299-luc2 cells are capable of colonizing and expanding within multiple anatomical sites overlapping with those commonly observed in advanced human NSCLC. In this model the frequency of bone metastases was higher than that of soft tissue metastases, and these were osteolytic, consistent with previous studies employing H1299 cell line administration via the intracardiac route [18, 19]. In contrast to these previous studies, we observed a comparatively higher incidence of bone metastasis as well as soft tissue metastasis involving adrenal glands, ovaries, and pancreas [18, 19].

We found that H1299-luc2 cells secrete chemokines (e.g. CXCL8, CCL5), growth factors (e.g. VEGF, PDGF-AB/BB, GM-CSF), and metalloproteinases (e.g. MMP-1, MMP-9). Expression of these factors has been reported in lung cancer patients and has been associated with advanced stage disease and/or a worse prognosis [20-23]. H1299-luc2 cells express multiple cell surface receptors (e.g. CXCR4, EGFR, u-PAR), exhibit constitutive activation of N-RAS, and they also lack PTEN activity. As a result, the cells show activation of key signal transduction pathways, including PI3K-AKT-mTOR, BRAF-MEK-MAPK, and STAT3 that have roles in promoting tumor growth, survival, and metastasis [24]. One of the key features for successful progression of metastasis is the ability of cancer cells to invade and adhere to ECM proteins. The ability of H1299-luc2 cells to adhere to ECM proteins such as fibronectin, collagen and laminin indicated that the cells expressed multiple integrins [25-28]. In addition, the invasive ability of H1299-luc2 cells may be associated with expression of epithelial to mesenchymal transition-associated markers (e.g. N-cadherin, and vimentin), plausibly supporting their propensity for metastatic inasiveness and eventual colonization. The expression of plethora of metastasis-related surface, chemokine/cytokines and signal transduction proteins by H1299-luc2 cells supported the potential of this metastasis model for the testing of molecular targeted therapies.

Interestingly, despite the fact that the H1299 cells express CXCR4, inhibition of this receptor failed to block metastatic colonization by H1299-luc2 cells. Suggesting that CXCL12 may have been acting acting as a trophic factor, we found that CXCR4 inhibition slowed the growth of H1299 metastases, a finding consistent with previous reports [29, 30]. However, at the dosage used, AMD3100 treatment did not appear to enhance survival. A similar observation was made in a breast cancer lung metastasis model, where neither AMD3100, nor knock-down of CXCR4 led to increased survival [29]. It would be of interest to investigate the effects of CXCR4 inhibition in a NSCLC model system that recapitulates the entire spectrum of the metastatic process, whereby distant lesions originate from a primary subcutaneous or orthotopic site. There are examples where an agent has discordant effects depending on whether treatment is directed at the primary site versus advanced metastatic disease [31]. However, at least in the type of immuno-compromised mice used in this study, we were unable to generate orthotopic lesions.

Herein, we describe the development and application of a NSCLC experimental metastasis model that may be particularly useful for the assessment of therapies aimed at advanced disease. We hypothesize that in contrast to preclinical models that rely drug testing in subcutaneous tumors, the use of more clinically-relevant preclinical models may be able to significantly improve the success rate of new agents under consideration for use in human clinical trials.

## MATERIAL AND METHODS

### Animals

Female, 5-6 wk old, NIH-III (*nu/nu; beige/beige*) mice were purchased from Charles River Canada. All animal studies were performed in compliance with Canadian Council of Animal Care guidelines, and with ethics approval from the University of Calgary Animal Care Committee.

### Cells and bioluminescence imaging

NCI-H1299 cells, obtained from the ATCC, were cultured in RPMI-1640, plus10% FBS, incubated at 37 oC, in 5% CO_2_. H1299 cells were transfected with a CMV promoter EGFP-luc2 fusion cDNA plasmid-based expression vector [32] using Lipofectamine 2000 (Invitrogen) and selection with G418 (1 mg/ml). This polyclonal population of cells were enriched for the EGFPhi (and hence, luc2^hi^) by FACSsorting as previously reported [32]. Luciferase activity was measured by seeding different number of cells in 96-well plates followed by the addition of D-luciferin and the plate was imaged on and IVIS Lumina (Caliper) imaging system.

### Molecular arrays

For 65-multiplex cytokine/chemokine assay, H1299-luc2 cells (2×10^6^/plate) were grown for 24 h and for MMP assay, cells were grown for 24 and 48 h. Supernatants were subjected to a human 65-plex cytokine and 8-plex MMP Discovery Array (EVE Technologies, Calgary, Alberta). Reverse-phase protein array (RPPA) carried out on cell lysates was performed by the RPPA Core Facility - Functional Proteomics (MD Anderson Cancer Center).

### Cell viability, adhesion and invasion assays

For cell viability assay, H1299-luc2 cells were seeded at 5000 cells per well in presence and absence 2.5 and 5μg/ml of AMD3100. Viability was measured after 48 h by using MTT. Cell adhesion assay was carried out using the CytoSelect™ ECM Cell Adhesion Assay kit (Cell BioLabs) following the instruction manual. The chemo-invasion assay was performed using BD BioCoat matrigel invasion chambers (catalog #354480) as described earlier, except 40,000 cells/well was used here [11].

### Experimental metastasis model and *in vivo* bioluminescence imaging

To generate metastasis H1299-luc2 cells (4×10^5^, 8×10^5^ per mouse) were injected into the left ventricle of anesthetized (1.5-2% isofluorane) as previously described [33]. For tail vein injections, mice were anaesthetized with 1.5-2% isofluorane, and then H1299-luc2 cells (4×10^5^ per mouse) were injected *via* the lateral tail vein. Growth of H1299-luc2 metastases was monitored via bi-weekly BLI as previously described [33].

### Drug treatment

AMD3100 (Sigma-Aldrich) was dissolved in sterile PBS and mice were administered 5 mg/kg of AMD3100 daily (control group of mice received PBS) for 5 wks (6 days per wk) subcutaneously, starting on the day of H1299-luc2 cell inoculation.

### Micro-computed tomography

To obtain qualitative assessments of bone osteolysis caused by metastasis, micro-computed tomography (μCT40, Scanco Medical) was used as previously described [32, 34]. To examine angiogenesis of a metastatic site, mouse were terminally perfused with a radio-opaque silicone rubber (Microfil, Flow Tech) to form a radio-opaque vascular cast that can be visualized using μCT as we have previously described [35].

### Histopathology

Following ex-vivo bioluminescence imaging, organs were fixed in 10% formalin. Skeletal tissues were fixed in 4% paraformaldehyde (PFA) for 1 wk, followed by decalcification in 14% EDTA for 2 wks. Tissues were then paraffin-embedded prior to sectioning and hematoxylin and eosin (H&E) staining.

## SUPPLEMENTARY MATERIAL FIGURES


