# Supplementary material for: Characterization of a murine model of metastatic human non-small cell lung cancer and effect of CXCR4 inhibition on the growth of metastases
Source: Oncoscience. 2015 Feb 9;2(3):263–71. doi: 10.18632/oncoscience.117 (PMC4394132; doi:10.18632/oncoscience.117)
Supplement: Supplementary file 1 [file oncoscience-02-0263-s001.pdf]

Characterization of a murine model of metastatic human non-small cell lung cancer and effect of CXCR4 inhibition on the growth of metastases

Supplementary Material

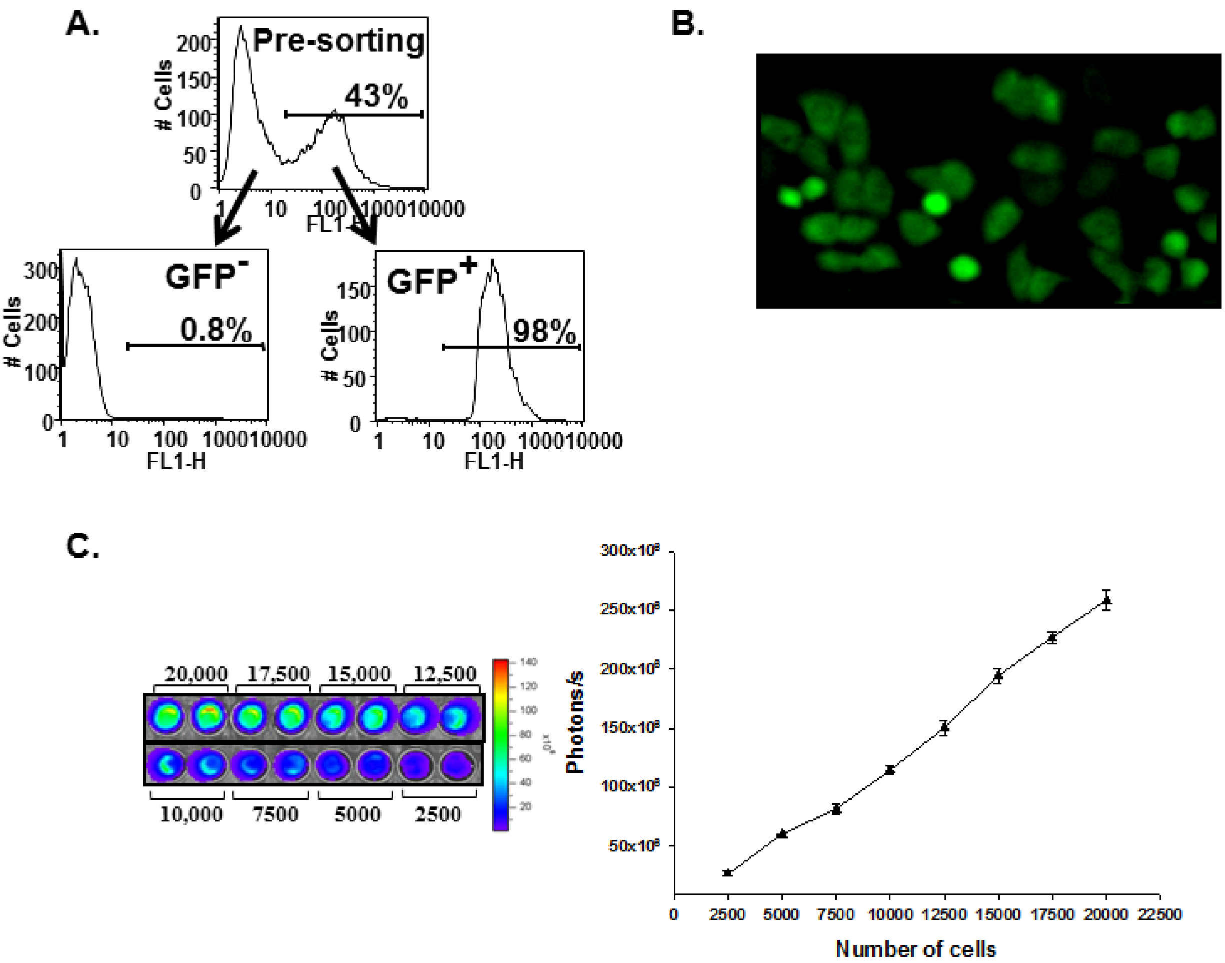

Figure S1: Establishing the polyclonal population of H1299-luc2 cells. (**A**) FACSorting of CMV-EGFP-luc2 vector transfected cells to obtain $EGFP^{hi}$ population. (**B**) A fluorescence microscopy image showing EGFP expression by H1299-luc2 cells (**C**) *In vitro* bioluminescence quantification of H1299-luc2 cells, showing correlation between photon emission rates and cell number, plotted as mean photons/sec/well ±SEM.

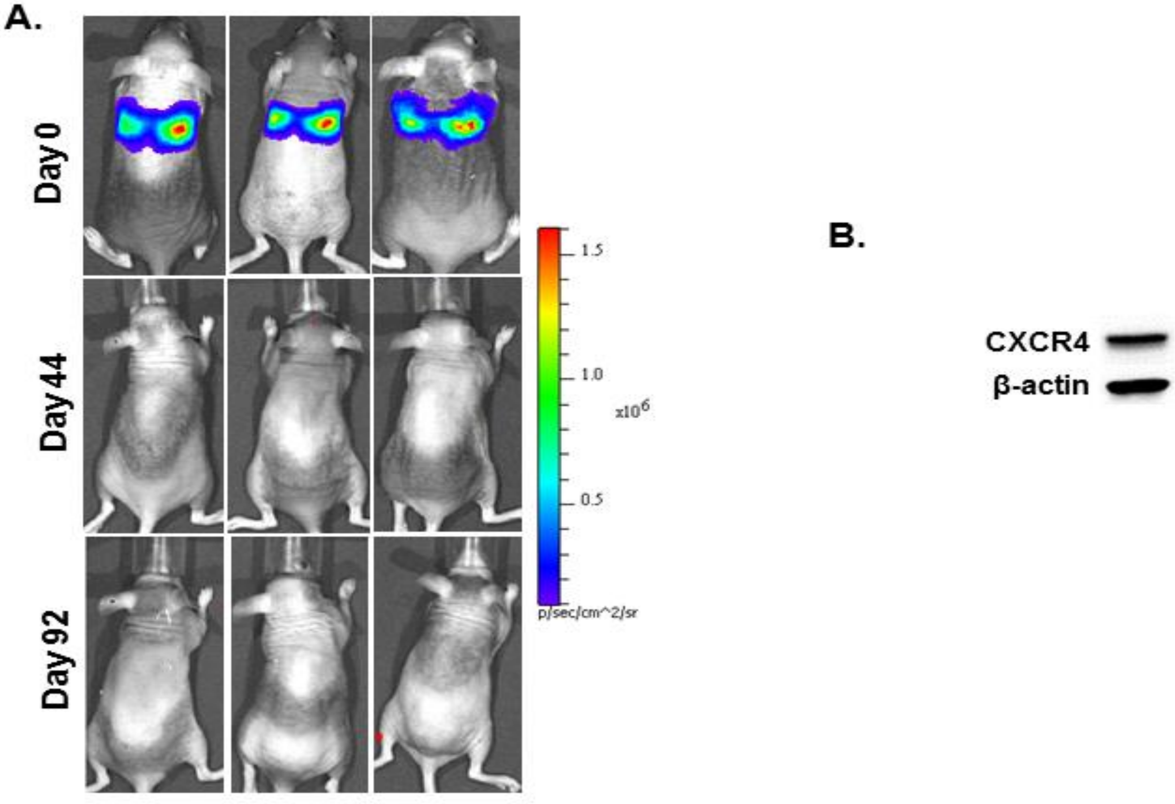

Figure S2: H1299-luc2 cells did not yield lung metasases following tail vein injection. (**A**) H1299-luc2 cells were injected into the tail veins of NIH-III mice, a representative bioluminescence image (dorsal day 0) showing a successful intravenous injection. Representative BLI images for three different mice taken at days 44, and 92 showing the absence of lung tumor development (n=5) following tail vein injection of H1299-luc2 cells. The day 0 time point shows that the tail vein injections were successful. (**B**) Immunoblot image showing CXCR4 expression in H1299-luc2 cells.
